# The correlation between serum magnesium, phosphorus, and calcium levels during pregnancy and the risk of gestational diabetes mellitus as well as adverse pregnancy outcomes

**DOI:** 10.3389/fcdhc.2026.1939424

**Published:** 2026-09-15

**Authors:** Achou Su, Jie Zhang, Xizhenzi Fan, Wenhui Song, Mingwei Li, Tianxiao Yu, Jin Zhang

**Affiliations:** 1 Research Center for Clinical Medical Sciences, The Fourth Hospital of Shijiazhuang, Shijiazhuang, China; 2 Department of Clinical Laboratory, The Fourth Hospital of Shijiazhuang, Shijiazhuang, China; 3 Medical Affairs Department, The Fourth Hospital of Shijiazhuang, Shijiazhuang, China; 4 Department of Anesthesiology, The Fourth Hospital of Shijiazhuang, Shijiazhuang, China

**Keywords:** calcium, gestational diabetes mellitus, magnesium, phosphorus, pregnancy outcome

## Abstract

**Introduction:**

This retrospective cohort study included 700 pregnant women who underwent antenatal check-ups and delivered at the Fourth Hospital of Shijiazhuang from January 2022 to December 2023, aiming to investigate the associations of second°trimester serum magnesium, phosphorus, and calcium with adverse pregnancy outcomes including gestational diabetes mellitus (GDM), anemia, and preterm birth. Mineral indicators were measured at 24–28 weeks of gestation, and baseline data were collected.

**Methods:**

Mann-Whitney U test was adopted to compare the serum mineral levels among women with different pregnancy outcomes, multivariable logistic regression and ROC curve analysis was utilized to further explore the correlations.

**Results:**

After adjusting for potential confounders, magnesium, phosphorus, and calcium levels were correlated with GDM risk, with magnesium and calcium exerting statistically significant protective effects against GDM while high phosphorus serving as a statistically significant GDM risk factor. High calcium was also negatively associated with the risks of anemia and preterm birth whereas high phosphorus was positively linked to preterm birth risk.

**Discussion:**

These findings demonstrate that second-trimester serum magnesium, phosphorus, and calcium levels are associated with GDM, providing clinical evidence for glycemic control interventions and individualized nutritional guidance during pregnancy.

## 1 Introduction

Gestational diabetes (GDM) is a common pregnancy complication with a high incidence rate. If not properly controlled, it can pose short-term or long-term potential health threats to both the mother and the fetus (1). Therefore, it is crucial to identify the risk factors for gestational diabetes and to clarify the necessity of early diagnosis. Current epidemiological studies and clinical trials suggest that maternal age, body mass index (BMI), and family history of diabetes may be potential susceptibility factors for gestational diabetes (2, 3). However, there are relatively few reports on the impact of minerals levels in pregnant women on GDM and related adverse pregnancy outcomes.

Previous studies have shown that mineral deficiencies, especially calcium and magnesium deficiency, may potentially contribute to the development of GDM (4–6). The presence of these minerals is essential for maintaining normal physiological functions and has been demonstrated to affect glucose metabolism and insulin sensitivity (7). Albaker, WI et al. reported that supplementing desalinated drinking water with magnesium improves metabolic parameters and insulin sensitivity in individuals with type 2 diabetes (8), furthermore, Liwei Luo et al. also found that magnesium supplementation during pregnancy can lower the fasting blood glucose of patients with GDM (9). Wu et al. revealed a U-shaped correlation between dietary phosphorus intake and the incidence of new-onset diabetes in the Chinese population (10), while its association with GDM remains unreported. A recent study has demonstrated that supplementation with vitamin D and calcium significantly reduces fasting blood glucose and serum insulin levels in patients with GDM (11). However, the specific relationship between serum levels of these minerals during pregnancy and the risk of developing GDM remains unclear.

Therefore, this study mainly aims to explore the relationship between the levels of calcium, magnesium, and phosphorus in the serum of pregnant women during the second trimester and the risk of GDM, while further analyzing the correlations of these indicators with other adverse pregnancy outcomes of the pregnant women and the newborns. We hope to clarify the potential roles of these minerals in GDM, identify new targets for prevention and management, and ultimately improve the health outcomes of both mothers and infants. This study also contributes to a more comprehensive understanding of the complex interplay between mineral metabolism during pregnancy and blood glucose stability, providing a basis for the development of new intervention strategies, thereby reducing the adverse pregnancy risks associated with GDM and better protecting the health of pregnant women and newborns.

## 2 Materials and methods

### 2.1 Study participants

This retrospective cohort study analyzed existing medical records of 700 pregnant women who delivered at The Fourth Hospital of Shijiazhuang, Hebei Province, China, between January 2022 and December 2023, enabled comprehensive data collection while avoiding additional patient recruitment or intervention. Serum mineral testing and GDM OGTT screening were conducted concurrently at 24–28 weeks gestation, before any dietary adjustment or hypoglycemic therapy, with all data retrospectively analyzed. Simultaneously, baseline demographic information of pregnant women was extracted from routine electronic obstetric medical records. This study was approved by the Ethics Committee of The Fourth Hospital of Shijiazhuang (Approval No. 20210077). All participating researchers collected patient data in accordance with ethical requirements. Inclusion criteria were as follows: (1) participants aged between 18 and 45 years old; (2) singleton pregnancy; (3) no other metabolic diseases and pregnancy complications; (4) clear-mindedness and willingness to participate and cooperate with the study; (5) prenatal care and delivery were completed in The Fourth Hospital of Shijiazhuang with complete data available. Exclusion criteria were as follows: (1) abnormal glucose tolerance or diabetes history before pregnancy; (2) abortion or bleeding during delivery; (3) thyroid disease, liver and kidney disease, immune system disease, or polycystic ovary syndrome.

**Figure 1:**
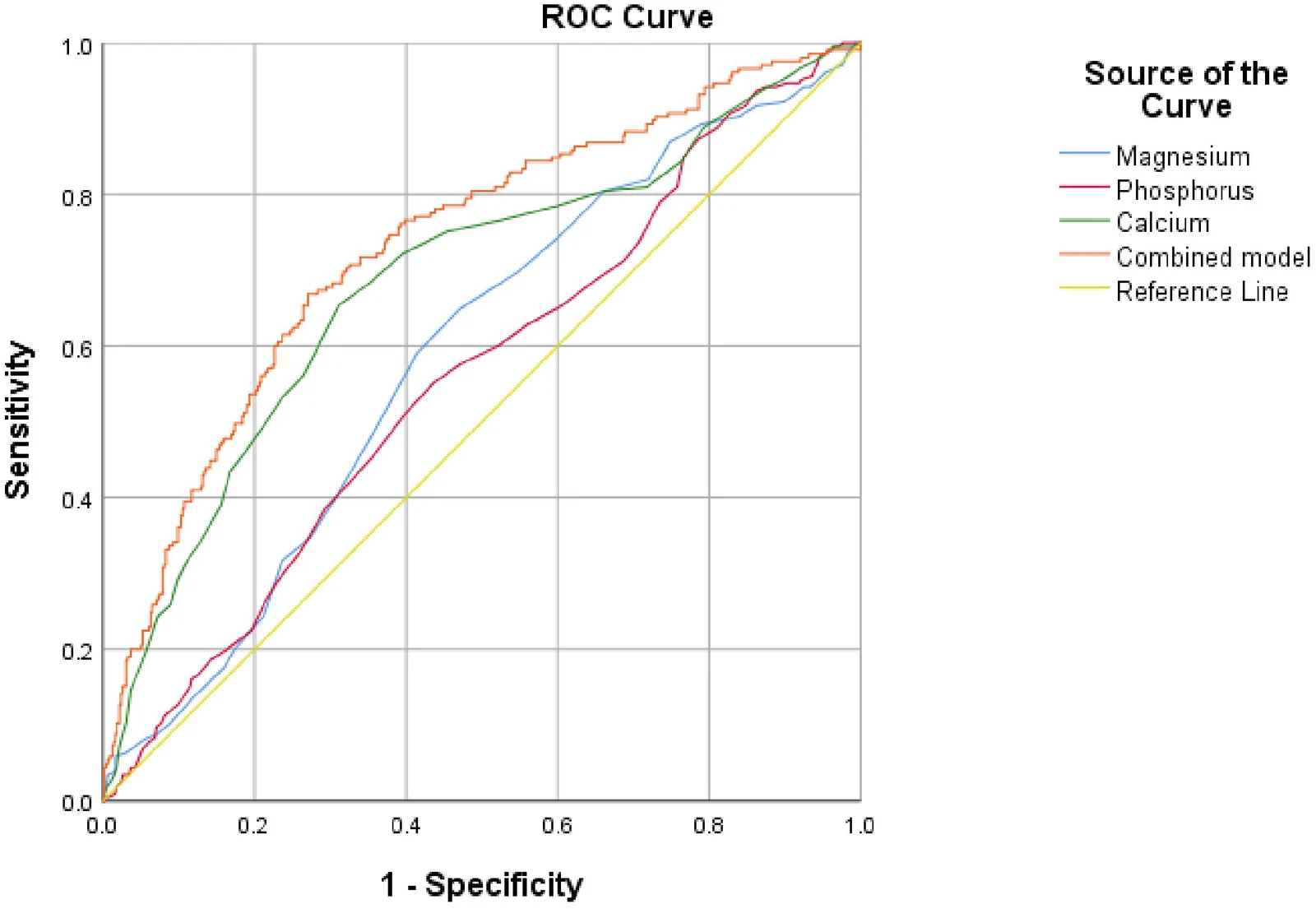
ROC curves of second-trimester serum magnesium, phosphorus, calcium and the combined model for predicting GDM. AUC: magnesium(blue) = 0.588; phosphorus(pink) = 0.559; calcium(green) = 0.684; combined model(orange) = 0.733.

### 2.2 Data collection

The basic details of each case were obtained through questionnaire surveys, including age, pre-pregnancy body mass index, parity, gestational age at delivery, education level, annual household income, history of adverse pregnancy outcomes and family history of diabetes. Data on pregnancy outcomes were obtained through a comprehensive review of clinical records. Blood samples were collected from pregnant women at 24-28 weeks gestation as part of their routine obstetric examination, drawn in the morning after fasting for at least 8 hours, and immediately centrifuged at 3000 r/min (12). Serum was then analyzed using an automated biochemical analyzer (Cobas 701, Roche). The serum mineral calibrators used were all proprietary to Roche, ensuring compatibility with the used reagent kits. The calibration mode employed multipoints calibration, and the comprehensive concentration-signal curves were established. The results of calcium, magnesium, and phosphorus tests from the obtained samples were collected. Only total calcium was recorded in historical medical records, while complete serum albumin data were unavailable for all participants; thus, albumin-corrected calcium and ionized calcium could not be calculated in this study. In addition, standardized records of antenatal calcium, magnesium, vitamin D and composite mineral supplements were not systematically archived in electronic medical files.

### 2.3 Diagnosis

Diagnosis of GDM: Excluding a history of impaired glucose tolerance or diabetes before pregnancy. GDM was diagnosed when blood glucose levels met at least 1 of the following criteria: fasting plasma glucose ranging from 5.1 to 6.9 mmol/L, a plasma glucose level of 10 mmol/L or higher after 1 hour, or a plasma glucose measurement between 8.5 and 11 mmol/L after 2 hours (13).

Diagnosis of anemia: Anemia severity in pregnant women is classified according to hemoglobin concentrations: 100 to less than 110 g/L indicates mild anemia, 70 to less than 100 g/L indicates moderate anemia, and less than 70 g/L indicates severe anemia (14).

Diagnosis of preterm birth: Delivery before 37 weeks of gestation is defined as preterm birth (15).

Pre-Pregnancy body mass index (BMI) classification: Pre-pregnancy weight was obtained from pregnant women during preconception visits, and BMI was calculated by dividing weight (kg) by height squared (m^2^), with results rounded to one decimal place. According to the Guidelines for the Prevention and Control of Overweight and Obesity in Chinese Adults, maternal pre-pregnancy BMI was categorized into two groups: a normal BMI group (BMI < 24.0) and an overweight/obesity group (BMI ≥ 24.0) (16).

Diagnosis of other pregnancy outcomes:

Premature rupture of membranes (PROM) was defined as spontaneous rupture of the fetal membranes before the onset of labor, in accordance with the American College of Obstetricians and Gynecologists (ACOG) guidelines (17). Chorioamnionitis was diagnosed clinically in the presence of maternal fever (≥ 39.0 °C, or ≥ 38.0 °C with at least one additional risk factor including fetal tachycardia, maternal leukocytosis, uterine tenderness, or purulent amniotic fluid), as recommended by ACOG (18). Preeclampsia was defined as new-onset hypertension after 20 weeks of gestation, with or without proteinuria, accompanied by maternal organ dysfunction, according to the ACOG 2013 (19). Macrosomia was defined as birth weight ≥ 4000 g, irrespective of gestational age. Low birth weight was defined as birth weight < 2500 g, regardless of gestational age, according to the World Health Organization (WHO) (20). Neonatal respiratory distress syndrome was diagnosed based on the presence of progressive respiratory failure shortly after birth, requiring supplemental oxygen or respiratory support (20).

### 2.4 Statistical analysis

Statistical analyses were performed using IBM SPSS Statistics version 26 (SPSS 26.0). For continuous variables, independent-sample t-tests were used if the data were normally distributed and had equal variance; results were presented as the mean ± standard deviation. If the data were not normally distributed, the Mann-Whitney U test was used, and results were reported with the interquartile range. Categorical variables were analyzed using the chi-square test and presented as frequency (n) and percentage (%). After conversion to per 0.1 mmol/L increment of serum minerals binary logistic regression was used to examine the relationships between serum mineral levels and adverse pregnancy outcomes. In addition, multivariable logistic regression models were applied to adjust for potential confounders. Adjusted odds ratios (OR) were calculated to assess the associations between trace element status and the risk of adverse pregnancy outcomes, with 95% confidence intervals (CI) used to show the precision of the estimates. A p-value < 0.05 was considered statistically significant.

## 3 Results

### 3.1 Baseline characteristics

According to the eligibility criteria, a total of 700 pregnant women were included, of whom 571 presented with at least one adverse pregnancy outcome (including gestational diabetes mellitus, anemia, premature rupture of membranes, chorioamnionitis, preeclampsia, macrosomia, low birth weight, preterm birth, and neonatal respiratory distress syndrome), while the remaining 129 participants had no gestational complications or abnormal delivery outcomes. **Table 1** presents the general characteristics which may be associated with GDM or adverse pregnancy outcomes. There was no significant difference between GDM or adverse pregnancy outcomes group and non GDM or adverse pregnancy outcomes group in baseline characteristics (p>0.05).

**Table 1:** Baseline information of patients (n=700).

Variables	Total n (%)	no GDM or adverse pregnancy outcomes n(%)	GDM or adverse pregnancy outcomes n(%)	χ^2^	*p*
Maternal age < 30 (years)	397 (56.7)	69 (53.5)	328 (57.4)	0.670	0.413
Maternal age ≥ 30 (years)	303 (43.3)	60 (46.5)	243 (42.6)		
Preconceptional BMI < 24 (kg/m^2^)	479 (68.4)	95 (73.6)	384 (67.3)	1.991	0.158
Preconceptional BMI ≥ 24 (kg/m^2^)	221 (31.6)	34 (26.4)	187 (32.7)		
Years of education ≤15	388 (55.4)	73 (56.6)	315 (55.2)	0.086	0.769
Years of education >15	312 (44.6)	56 (43.4)	256 (44.8)		
Family annual income <100 000 yuan	380 (54.3)	69 (53.5)	311 (54.5)	0.041	0.840
Family annual income ≥100 000 yuan	320 (45.7)	60 (46.5)	260 (45.5)		
Family history of diabetes (none)	644 (92.0)	121 (93.8)	523 (91.6)	0.694	0.405
Family history of diabetes (yes)	56 (8.0)	8 (6.2)	48 (8.4)		
Adverse pregnancy history (none)	519 (74.1)	92 (71.3)	427 (74.8)	0.657	0.418
Adverse pregnancy history (yes)	181 (25.9)	37 (28.7)	144 (25.2)		
Primipara	470 (67.1)	80 (62.0)	390 (68.3)	1.885	0.170
Multipara	230 (32.9)	49 (38.0)	181 (31.7)		

The Chi-square test was used to analyze the continuous variables between the 2 groups. and categorical data were displayed as frequencies (n) and percentages (%); BMI, Body Mass Index; GDM, gestational diabetes mellitus.

### 3.2 Comparison of serum magnesium, phosphorus, and calcium levels between groups

In **Table 2**, the serum magnesium, phosphorus, and calcium levels corresponding to different outcomes are represented by the median (P25 - P75). There were significant differences in serum magnesium, phosphorus and calcium levels between GDM group and non GDM group (p<0.05). Additionally, there were also differences in calcium levels in anemia and preterm birth (p<0.05); specifically, lower serum calcium levels were associated with increased risk of adverse pregnancy outcomes. Furthermore, there was a significant difference in phosphorus levels between preterm delivery (p<0.05). These data implied that the serum levels of magnesium, phosphorus and calcium might be related to adverse pregnancy outcome.

**Table 2:** Magnesium, phosphorus, and calcium levels in different pregnancy outcomes (n=700).

Pregnancy outcome		N	Magnesium (mmol/L)	*Z*	*p*	Phosphorus(mmol/L)	*Z*	*p*	Calcium(mmol/L)	*Z*	*p*
GDM	Yes	205	0.71 (0.67-0.75)	-3.662	<0.001	1.28 (1.19-1.34)	-2.443	0.015	1.99 (1.90-2.06)	-7.692	<0.001
	No	495	0.73 (0.68-0.78)			1.25 (1.17-1.32)			2.06 (2.00-2.10)		
Anemia	Yes	343	0.72(0.67-0.78)	-1.396	0.163	1.25 (1.17-1.32)	-1.753	0.080	2.03 (1.95-2.08)	-4.484	<0.001
	No	357	0.72(0.67-0.76)			1.26 (1.19-1.33)			2.06 (1.99-2.11)		
PROM	Yes	181	0.71 (0.66-0.76)	-1.692	0.091	1.26 (1.17-1.33)	-0.179	0.858	2.04 (1.95-2.10)	-1.093	0.275
	No	519	0.72 (0.68-0.77)			1.25 (1.18-1.32)			2.05 (1.97-2.10)		
Chorioamnionitis	Yes	115	0.72 (0.67-0.77)	-0.526	0.599	1.25 (1.15-1.33)	-0.742	0.458	2.04 (1.96-2.09)	-0.524	0.600
	No	585	0.72 (0.67-0.76)			1.26 (1.18-1.33)			2.05 (1.97-2.10)		
Preeclampsia	Yes	43	0.74 (0.70-0.78)	-1.724	0.085	1.28 (1.20-1.36)	-1.477	0.140	2.03 (1.94-2.07)	-1.501	0.133
	No	657	0.72 (0.67-0.77)			1.25 (1.17-1.32)			2.05 (1.97-2.10)		
Fetal weight	Normal	631	0.72 (0.67-0.76)			1.25 (1.17-1.32)			2.05 (1.97-2.10)		
	Macrosomia	29	0.75 (0.69-0.79)	-1.087	0.277^a^	1.26 (1.17-1.39)	-0.621	0.534^a^	2.06 (1.98-2.11)	-0.513	0.608^a^
	Low weight	40	0.73 (0.69-0.79)	-1.341	0.180^b^	1.26 (1.20-1.34)	-0.360	0.719^b^	2.04 (1.93-2.09)	-1.096	0.273^b^
Preterm birth	Yes	50	0.72 (0.68-0.77)	-0.034	0.973	1.28 (1.23-1.38)	-2.606	0.009	2.03 (1.89-2.07)	-2.399	0.016
	No	650	0.72 (0.67-0.77)			1.25 (1.17-1.32)			2.05 (1.97-2.10)		
Neonatal respiratory distress	Yes	26	0.72 (0.68-0.75)	-0.282	0.778	1.31(1.22-1.35)	-1.907	0.056	2.04 (1.97-2.10)	-0.176	0.860
	No	674	0.72 (0.67-0.77)			1.25 (1.17-1.32)			2.05 (1.97-2.10)		

The Mann-Whitney U test was used to analyze the continuous variables between the 2 groups. Continuous variables are presented as interquartile range; GDM, gestational diabetes mellitus; PROM, premature rupture of membranes. *P*^a^:comparison between normal birth weight and macrosomia. *P*^b^:comparison between normal birth weight and low birth weight.

### 3.3 Associations between serum magnesium, phosphorus, and calcium levels and adverse pregnancy outcomes

To improve clinical interpretability of effect estimates, serum magnesium, calcium and phosphorus were transformed to represent per 0.1 mmol/L increments by multiplying original values by 10, and relevant adjusted odds ratios are shown in revised **Table 3**. After adjusting for potential confounders (age, preconceptional BMI, family history of diabetes, adverse pregnancy history and parity), magnesium, phosphorus, and calcium were directly correlated with the risk of GDM, where magnesium and calcium functioned as protective factors (OR = 0.718, 95%CI: 0.576-0.896, p=0.003; OR = 0.484, 95%CI=0.400-0.585, p<0.001), while phosphorus served as a risk factor (OR = 1.145 95%CI=1.016-1.290, p=0.027). The findings also indicated that calcium was negatively associated with the risk of anemia (OR = 0.729 95%CI=0.618-0.860, p<0.001). Phosphorus was positively related to the risk of preterm birth, and calcium was negatively related to the risk of preterm birth (p<0.05 in both instances).

**Table 3:** Univariate and multiple regressions between serum magnesium, phosphorus, and calcium levels and adverse pregnancy outcomes (n=700).

	GDM			Anemia			preterm birth		
	OR	95%CI	*p*	OR	95%CI	*p*	OR	95%CI	*p*
Magnesium									
Unadjusted	0.685	0.550-0.851	0.001	1.172	0.976-1.408	0.089	1.225	0.906-1.655	0.187
Adjusted	0.718	0.576-0.896	0.003	1.168	0.969-1.409	0.103	1.254	0.937-1.678	0.127
Phosphorus									
Unadjusted	1.173	1.044-1.318	0.007	0.931	0.841-1.030	0.164	1.343	1.082-1.667	0.007
Adjusted	1.145	1.016-1.290	0.027	0.959	0.864-1.064	0.430	1.403	1.118-1.762	0.004
Calcium									
Unadjusted	0.485	0.403-0.583	<0.001	0.724	0.615-0.852	<0.001	0.653	0.486-0.878	0.005
Adjusted	0.484	0.400-0.585	<0.001	0.729	0.618-0.860	<0.001	0.687	0.501-0.927	0.014

Binary logistic regression was employed. Adjusted for age, preconceptional BMI (continuous variable), family history of diabetes, adverse pregnancy history and parity. OR expressed per 0.1 mmol/L increase in serum mineral concentration. OR, odds ratio; BMI, body mass index; GDM, gestational diabetes mellitus; PROM, premature rupture of membranes.

### 3.4 ROC curve analysis was used to assess the predictive performance of magnesium, phosphorus, calcium, and their combined model for GDM

ROC curve analysis was performed to evaluate the predictive value of serum magnesium, phosphorus, calcium, and their combined logistic regression model for GDM. Among the three mineral elements, calcium exhibited the highest discriminative ability, with an AUC of 0.684 (95% CI: 0.639–0.730, p<0.001). At the optimal cut-off value of 2.025, it yielded a sensitivity of 65.4% and a specificity of 68.9%. Magnesium showed an AUC of 0.588 (95% CI: 0.542–0.633, p<0.001), with an optimal cut-off of 0.725, corresponding to a sensitivity of 64.9% and a specificity of 52.9%. Phosphorus demonstrated the lowest individual predictive value, with an AUC of 0.559 (95% CI: 0.512–0.605, p=0.015). At the cut-off value of 1.265, the sensitivity and specificity were 55.1% and 56.4%, respectively. In contrast, the combined model had the highest AUC of 0.733 (95% CI: 0.691–0.775, p<0.001). Using the maximum Youden index, the optimal cut-off probability was determined as 0.301, at which point the sensitivity and specificity reached 66.8% and 72.9%, respectively. This combined model exhibits good discriminative ability, suggesting that integrating these three indicators provides superior clinical risk stratification.

## 4 Discussion

Some minerals in the human body play indispensable and critical roles. In the reproductive system, they participate in hormone regulation, significantly affect endometrial function, ovarian function and ovulation, and are also closely related to oxidative stress (21). Adequate mineral concentrations during pregnancy are essential not only for maternal health but also for normal fetal growth and development (22, 23). This study mainly investigated the associations between maternal serum levels of magnesium, phosphorus and calcium in the second trimester and gestational diabetes mellitus. We also explored whether these serum mineral levels were related to other adverse birth outcomes, such as anemia and preterm birth. By comprehensively examining these relationships, we aimed to provide evidence for nutritional intervention and early warning, with the ultimate goal of reducing adverse pregnancy outcomes related to gestational diabetes and improving maternal and neonatal health.

We first compared the baseline data of the parturients with GDM or other adverse pregnancy outcomes with those of the unaffected population. The results showed that there were no differences between the two groups (*p>*0.05). Subsequently, we studied the influences of magnesium, phosphorus, and calcium levels on the occurrence of GDM and other pregnancy outcomes. After adjusting for potential confounders (age, pre-pregnancy BMI, family history of diabetes, adverse pregnancy history, and parity), a direct correlation was identified between these minerals and the risk of GDM. Specifically, magnesium and calcium demonstrated a protective effect on GDM. This study revealed that women with higher magnesium and calcium concentrations had a lower risk of GDM. Similar to our findings, a recent meta-analysis revealed that, among European populations and individuals in the third trimester of pregnancy, patients with GDM exhibited lower circulating magnesium levels compared to their healthy counterparts (5). Liu et al.’s retrospective study revealed that calcium deficiency during early pregnancy is an independent risk factor for the development of GDM (4). This can be attributed to the fact that magnesium and calcium are capable of reducing insulin resistance and promoting insulin sensitivity, thereby promoting the stable control of blood glucose (24, 25). Magnesium may maintain normal glucose metabolism by participating in energy metabolism and protein synthesis (26), reduced calcium transport proteins and destroyed calcium signaling have been observed in GDM (27). Existing studies suggest that magnesium and calcium supplementation in early pregnancy can reduce the incidence of gestational diabetes mellitus, which is consistent with our finding that elevated serum magnesium and calcium concentrations in the second trimester are correlated with a lower risk of GDM. Combined supplementation of calcium and vitamin D is beneficial for stabilizing blood glucose in patients with GDM, magnesium supplementation upregulated the gene expression of glucose transporter 1 (GLUT1) in patients with GDM, thereby improving glucose and lipid metabolism (28, 29). Furthermore, low calcium is also a risk factor for preterm birth and anemia.Calcium deficiency may disturb calcium-channel-dependent myometrial function and increase the risk of preterm birth (30); meanwhile, calcium metabolic disorder may interact with iron homeostasis to exacerbate maternal anemia.

Contrarily, elevated phosphorus levels are considered to be a risk factor for GDM. A large prospective cohort study conducted in France has demonstrated that high phosphorus intake is associated with an increased risk of developing type 2 diabetes (31). However, the correlation between phosphorus and GDM is rarely reported. The report by A Gobaht, K. et al. revealed that high phosphorus levels are positively correlated with the occurrence of GDM (32). Elevated phosphorus levels may contribute to a higher risk of GDM through multiple biological mechanisms. For example, FGF-23, a phosphaturic hormone primarily secreted by osteocytes, plays a critical role in maintaining phosphate and vitamin D homeostasis. Elevated serum phosphate levels may impair glucose homeostasis by disrupting the FGF-23/Klotho signaling pathway (33, 34). Another literature reported that, excessive phosphorus promotes reactive oxygen species production, resulting in pancreatic β-cell apoptosis and reduced insulin secretion (35, 36).Given the limitations inherent in retrospective cohort studies and the limited number of existing cases, further prospective studies are warranted to validate this finding. In light of this, we postulated that excessive phosphorus in the body may disrupt the insulin signaling pathway, resulting in exacerbated insulin resistance and an increased risk of GDM. Furthermore, high phosphorus levels may also constitute a risk factor for preterm birth. The underlying biological mechanism may involve high phosphorus levels triggering the activation of the NLRP3 inflammasome, thereby promoting the initiation of labor (37, 38). Elevated serum phosphorus stimulates fibroblast growth factor 23 (FGF-23) secretion, suppresses the expression of the anti-aging protein Klotho, and consequently induces endothelial dysfunction, leading to vasoconstriction (39).

While this study provides valuable insights into the association between serum magnesium, phosphorus, and calcium levels and the risk of GDM and adverse pregnancy outcomes, several limitations inherent to the retrospective design should be acknowledged. First, historical electronic medical records lacked standardized records of antenatal mineral, vitamin D and combined nutritional supplements, which may introduce confounding bias into the correlation analysis between serum mineral concentrations and pregnancy complications. Second, only total serum calcium was measured without albumin correction; physiological gestational hypoalbuminemia may reduce total calcium concentrations independent of the level of biologically active ionized calcium. Third, serum mineral indicators were only tested once at 24–28 weeks of gestation, which cannot reflect dynamic changes of mineral metabolism throughout the whole pregnancy. Fourth, this observational study cannot establish definite causal relationships, although simultaneous blood collection and GDM screening largely reduced reverse causation bias.

## 5 Conclusions

This study revealed the association between the maternal serum magnesium, phosphorus, and calcium levels as related to the risk of GDM, suggesting that magnesium and calcium potentially play a protective role, whereas elevated phosphorus levels might constitute a risk factor for GDM. These findings have considerable significance for the prevention and management of GDM, particularly in nutritional guidance before and during the pregnancy. The present observational correlation suggests that sustained adequate serum magnesium and calcium status may correlate with lower GDM risk; however, randomized controlled trials are essential to verify whether targeted mineral supplementation brings clinical glycemic benefits, and no definitive supplementation recommendations can be drawn from this retrospective cohort study. Nevertheless, these conclusions need to be further verified in larger prospective studies to provide more reliable evidence for clinical practice.

## Data Availability

The original contributions presented in the study are included in the article/supplementary material, further inquiries can be directed to the corresponding author/s.
